# The Impact of Environmental Quality Dimensions and Green Practices on Patient Satisfaction from Students’ Perspective—Managerial and Financial Implications

**DOI:** 10.3390/healthcare13141673

**Published:** 2025-07-11

**Authors:** Nikola Milicevic, Nenad Djokic, Ines Djokic, Jelena Radic, Nemanja Berber, Branimir Kalas

**Affiliations:** 1 Faculty of Economics in Subotica, University of Novi Sad, Segedinski put 9-11, 24000 Subotica, Serbia; nikola.milicevic@ef.uns.ac.rs (N.M.); nenad.djokic@ef.uns.ac.rs (N.D.); ines.djokic@ef.uns.ac.rs (I.D.); nemanja.berber@ef.uns.ac.rs (N.B.); 2 Faculty of Medicine, University of Novi Sad, Hajduk Veljkova 3, 21000 Novi Sad, Serbia; jelena.radic@mf.uns.ac.rs

**Keywords:** environmental knowledge, environmental quality dimensions, green practices, patient satisfaction

## Abstract

**Background/Objectives**: Healthcare institutions, similar to other service providers, should prioritize their clients—in this case, patients—to effectively meet their needs. However, fulfilling this objective becomes increasingly challenging due to numerous factors. Therefore, this study explores student patient satisfaction by examining the effects of environmental quality dimensions (Internal Spaces, External Spaces, And Social Environment) and green practices, as well as investigating how environmental knowledge moderates the relationship between green practices and patient satisfaction. **Methods**: Given the latent nature of the variables investigated, structural equation modeling (SEM) was employed. Some variables were conceptualized as hierarchical constructs comprising higher-order and lower-order components. Before testing the relationships among variables, reliability and validity assessments were performed. For this purpose, the SmartPLS 4 software was used. Since the focus of the research was on students’ health in general, the sample consisted of 280 students from the University of Novi Sad (Republic of Serbia). **Results**: Among the three environmental quality dimensions, only the Social Environment had a significant and positive influence on patient satisfaction. Furthermore, the green practices emerged as a significant determinant of patient satisfaction. However, the moderating effect of environmental knowledge on this relationship was found to be non-significant. **Conclusions**: This research underscores the significance of patient satisfaction as a critical objective for healthcare institutions. Special attention should be directed toward enhancing positive interactions between medical staff and patients and adopting green practices. Consequently, certain managerial aspects related to human resource management (such as adequate staffing and organization of personnel) should be considered. In addition, issues concerning financial challenges and benefits regarding the implementation of green practices in healthcare were presented.

## 1. Introduction

Many recent changes, including the increase in world population, the larger number of chronic diseases, and the lack of choices regarding healthy living, have significantly impacted the healthcare industry [1]. Consequently, healthcare organizations face continuous pressure to provide high-quality medical services without compromising patient safety or incurring excessive costs [2]. The factor that makes their position even more complex refers to an expanding number of competitors at the local and international level [1].

To cope with such conditions, healthcare organizations need to reconsider their strategies [1] and concentrate on patient assessments, their suggestions, and experiences [3]. Hereby, the patient-centered approach has emerged as a way to encourage the delivery of higher-quality healthcare [4] and to enhance patient satisfaction [5]. According to Ross et al. [6], patient satisfaction may lead not only to success in retaining current patients and attracting new ones, but it may also contribute to the success of medical treatment. Patient satisfaction may even be considered as an imperative, or a critical issue [7].

The improvement of process should be in line with patients’ expectations, with a focus on those operational areas, which have quality issues [1]. Therefore, attention needs to be dedicated to all elements of a hospital environment, including both physical and social aspects. The analysis of information obtained from patients could be of great help in the decision-making process, bearing in mind that they represent the direct users of healthcare services. Therefore, all environmental elements should be examined from the aspect of patients’ perceptions, especially when it comes to their relations with patients’ satisfaction.

Additionally, given the importance of environmental issues as well as people’s increasing environmental awareness and positive attitudes toward ecology [8], this research includes an examination of green practices. Through their adoption, healthcare institutions may be viewed more favorably by patients, particularly younger individuals, who, according to Calculli et al. [8], are actively engaging with environmental issues. Nonetheless, environment-orientated sustainability represents one of the dimensions of sustainability practices in healthcare, along with customer, employee, and community-oriented sustainability [9]. In the context of sustainability management in healthcare organizations, Bosco et al. [10] considered the environmental aspect as one of the three pillars of the ESG approach; the other two were the social pillar (which included various stakeholders such as patients, employees, suppliers, and communities) and the governance pillar.

Green practices in hospitals “aim to increase the sustainability impact of their operations by integrating and coordinating sustainability practices with healthcare service and operational processes” ([11], p. 3). The implementation of these practices is essential due to healthcare’s significant environmental impact, particularly in terms of pollution—only in the United Kingdom, 18 million tons of carbon dioxide are emitted annually by the National Health Service (NHS); at the same time, in the United States, there was an increase of 6% in total gas emissions from healthcare organizations from 2010 to 2018 [12]. On a global level, the healthcare sector participates in total carbon dioxide emissions with 4.4% [13]; according to the World Health Organization, in high-income countries, the amount of hazardous healthcare waste can reach up to 0.5 kg per hospital bed each day [14]. Moreover, it contributes to other serious environmental concerns, such as high energy consumption and the production of substantial amounts of waste, including infectious and/or toxic materials [15]. Hence, there is an irony in the fact that a sector dedicated to healing patients also contributes to climate change, thereby worsening environmental health [16].

Building on insights from the global healthcare literature, this paper’s research objective is to examine patient satisfaction within the context of the healthcare institution’s environmental quality dimensions, with a specific focus on the student population. The importance of students’ health lies not only in the fact that it influences their learning potential [17] but also in the context of their future engagements—as potential significant public and business actors, their health-related lifestyles, attitudes, and beliefs may disproportionately impact the population’s health [18]. Additionally, health-associated habits formed during studies may be hard to change in older age [18]. In this regard, the research questions refer to assessing the impact of three environmental quality dimensions (Internal Spaces, External Spaces, and Social Environment) on patient satisfaction. Besides these quality-related aspects, the emphasis was on green practices, whose effect was moderated by environmental knowledge. Accordingly, the second research objective addresses the relationship between patients’ perception of green practices in the healthcare organization and their satisfaction. The corresponding research question seeks to identify the impact of perceived green practices on patient satisfaction.

After explaining all variables and their relations, a conceptual model was developed based on the PHEQI (Perceived Hospital Environment Quality Indicator) approach. A particular contribution of this model lies in its structural design, where three dimensions of the healthcare institution environment were structured as hierarchical constructs. Furthermore, the integration of green practices adds to the model’s originality. The results are followed by a discussion of managerial and financial implications.

## 2. Literature Review

For many decades, researchers have analyzed patient satisfaction, trying to explain this concept and/or examine its relations with other variables [19,20,21,22,23,24].

In the early 1980s, Linder-Pelz [19] defined patient satisfaction as positive evaluations associated with different dimensions of healthcare, where the subject of evaluation can be a clinical visit, the whole treatment, a specific healthcare surrounding, or the system at large. This research also resulted in five hypothesized antecedents of those positive ratings, including expectations, value, entitlement, occurrences, and interpersonal comparisons. Besides the previously mentioned value-expectancy model [19], there are some alternative approaches related to satisfaction, such as discrepancy theory, fulfillment theory, and equity theory [20]. In line with these alternatives, satisfaction was explained in the context of certain comparisons between individual desires and experiences, between desired and received rewards, and between inputs and outputs, respectively [20].

Later, in a number of studies, patient satisfaction was analyzed at the general level as a one-dimensional construct. In the research of Chakraborty et al. ([25], p. 154), patient satisfaction, defined as the “extent to which the patient’s feelings and cognitive assessment of service is positive”, was measured with the use of four items. For this purpose, Manzoor et al. [26] applied a nine-item scale, while Senić and Marinković [23] used three items based on the American Customer Satisfaction Index model.

### 2.1. Environmental Quality Dimensions

Patient satisfaction was examined in relation to other variables, among which was usually the service quality. As shown in the results of a number of studies, patient satisfaction was positively affected by several service quality dimensions—responsiveness, assurance, communication, and discipline [22]; and personal relationships, promptness, and tangibility [23]. One of the healthcare components that may impact patients’ overall satisfaction and well-being refers to the hospital’s environment, which can be expressed through the Environmental Quality Perception (EQP) construct [27]. Fornara et al. [28] developed Perceived Hospital Environment Quality Indicators (PHEQIs) as an approach for evaluating hospital settings. It includes four scales—three associated with physical environments (external spaces, care unit, and in-patient area/(out-patient) waiting area) and one related to a social environment, whereby within each scale several factors were extracted. The PHEQI instrument (its selected items or scales) has already been used in several studies [29,30,31,32,33,34,35], where, in some of them, the focus was on patient satisfaction as well. Hence, in research devoted to a hospice as the specific type of healthcare, Manca et al. [34] found significant and positive correlations between most of the indicators of three hospice settings (internal common spaces, internal private spaces, and external spaces) and the overall users’ satisfaction. Bearing in mind previously mentioned studies, three hypotheses related to the institution’s physical (external and internal spaces) and social environments were tested:

**H1.**  *Perceptions of the quality of the healthcare institution’s external spaces positively affect patient satisfaction.*

**H2.**  *Perceptions of the quality of the healthcare institution’s internal spaces positively affect patient satisfaction.*

**H3.**  *Perceptions of the quality of the healthcare institution’s social environment positively affect patient satisfaction.*

### 2.2. Green Practices

In addition to the environmental quality dimensions, along with the increasing importance of sustainability agenda, attention has been paid to green healthcare practices. To the authors’ knowledge, only a few studies have examined their relationship with patient satisfaction. Chakraborty et al. [25] developed a model that included green practices, agile practices, care service delivery, and patient satisfaction. Hereby, under green practices, the previously mentioned authors mean the level up to which the hospital applies eco-friendly practices and conservation of non-renewable energy resources needed for the implementation of care delivery and care service activities. Following their results, green practices directly and positively affected care service delivery and agile practices, while the effect on patient satisfaction was also positive, but significant only indirectly.

The relationship between green operational practices and patient satisfaction was the subject of the analysis in the research of Mahrinasari et al. [36]. Moreover, they examined the effect of E-CRM on patient satisfaction as well as the moderating effects of green social influence. The research involved patients who received treatment (and whose satisfaction was measured) and hospital employees. Considering green operational practices, Mahrinasari et al. [36] presented them through several variables: green building, eco-design, green supply chain, and innovation, and all four of them positively affected patient satisfaction. The positive impact of green or sustainable practices on satisfaction was recorded in other service sectors as well [37,38,39,40]. In line with these results, hypothesis H4 was set:

**H4.**  *Perception of green healthcare practices positively affects patient satisfaction.*

The impact of green healthcare practices on patient satisfaction may depend on the level of environmental knowledge (a concept similar to “environmental literacy”), which “can be defined as a general knowledge of facts, concepts, and relationships concerning the natural environment and its major ecosystems” ([41], p. 48.) Although environmental knowledge and environmentally friendly behavior are distinct concepts, they can be closely related, as individuals with greater knowledge of environmental issues are more likely to engage in pro-environmental behavior [41]. The results of several research works pointed to the important role of environmental knowledge when it comes to people’s environmental-friendly attitudes, intentions, and/or behavior, expressed through its direct [42,43,44] and moderating effects [45,46,47]. Issock Issock et al. [48] found that environmental knowledge significantly moderated the influence of environmental value on green customer satisfaction by strengthening this effect.

In the context of healthcare, patients who are more knowledgeable about environmental issues may be more appreciative of sustainability initiatives undertaken in healthcare organizations. As a result, when such individuals perceive green healthcare practices, they may evaluate the institution more favorably, leading to greater satisfaction. Therefore, it was expected that environmental knowledge would influence the relationship between perceived green healthcare practices and patient satisfaction, which was tested through hypothesis H5:

**H5.**  *Environmental knowledge moderates the effect of perceived green healthcare practices on patient satisfaction.*

**Figure 1:**
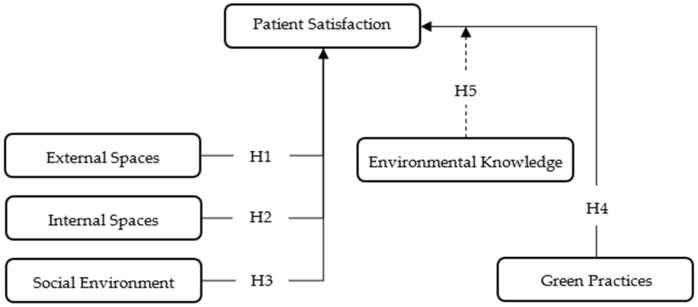
Conceptual model.

Main constructs and hypothesized relations are presented within the conceptual model in Figure 1. 

Unlike similar studies, this model includes both environmental quality dimensions and green practices as predictors of patient satisfaction, as well as the moderating role of environmental knowledge.

## 3. Research Methodology

### 3.1. Participants and Data Collection

The research uses a convenience sample of 280 students from the University of Novi Sad (the largest university in the Autonomous Province of Vojvodina, Republic of Serbia) who have used medical services at least once at the Institute for Student Health Protection in Novi Sad. Their mean age is 21.7 and the majority of them (more than 60%) were female students, which is understandable considering that in the Autonomous Province of Vojvodina, a greater number of female (compared to male) students enrolled in studies in the past few years [49,50,51]. Regarding the sample size, the approach based on the minimum R^2^ value was followed [52]. The endogenous construct with the highest number of incoming arrows was Internal Spaces, which was predicted by five latent variables (Lighting, Spatial Physical Comfort, Air Quality, Orientation (internal), and Quietness). Consequently, to detect an R^2^ of 0.25 with 80% statistical power at the 5% significance level, a minimum of 45 respondents is required, which is considerably below our sample size.

In the Institute for Student Health Protection in Novi Sad, as in any other state-run student polyclinics in Serbia, medical services are free for students. Although that is the reason why Serbian students usually rely on those services and rarely visit private clinics, further increases in the students’ living standard may lead to changes in their behavior regarding the choice of healthcare services provider [23]. Keeping this in mind, as well as the relevance of the development of relationships and trust between healthcare organizations and their patients, attention should be paid to students’ satisfaction concerning the provided medical services and its main factors [23].

For collecting data, we applied the online questionnaire. The survey was conducted anonymously, and participants were explicitly informed of this in advance. Participation was voluntary, and students were invited to take part in the study by professors from the University of Novi Sad. The questionnaire was administered via an online platform accessible to both professors and students, which has already been used for information and material exchange.

### 3.2. Instruments

To measure all constructs, four different scales were used: one for environmental quality dimensions, one for green practices, one for environmental knowledge, and one for patient satisfaction. Each scale consisted of several statements, which students evaluated using a 5-point Likert scale ranging from 1 (totally disagree) to 5 (totally agree).

When it comes to the environmental quality dimensions, we relied on the PHEQI questionnaire [27], which consists of both positively and negatively formulated items. However, as the Institute for Student Health Protection in Novi Sad is an integral part of the university campus, located within the complex of the student dormitory, there are certain limitations regarding the analysis of its environment. Additional adaptations of the original instrument were performed in accordance with the characteristics of the Institute. Hence, we adapted the PHEQI approach and applied the hierarchical modeling by setting the Social Environment, External Spaces, and Internal Spaces as higher-order constructs determined by their corresponding lower-order constructs.

Two constructs—Care for Social and Organizational Relationship and Privacy—formed the Social Environment. Care for Social and Organizational Relationship was measured by seven items associated with a welcome from the staff (CSOR1), doctors’ understanding toward patients (CSOR2), nurses’ understanding toward patients (CSOR3), the provision of information related to medical examinations, therapies, and interventions (CSOR4), cooperative atmosphere among staff members (CSOR5), institute organization (CSOR6), and asking information (CSOR7)). Privacy was measured by four items associated with talking to the staff about delicate issues (P1), the ease of identifying the staff’s name, surname, and function (P2), the crowd (P3), and people’s privacy (P4).

Three constructs—Upkeep and Care, Building Aesthetics, and External Orientation—formed External Spaces. Upkeep and Care was measured by five items associated with building condition (UC1), its entrance (UC2), paths and sidewalks’ condition (UC3), external space maintenance (UC4), and cleanliness (UC5). Building Aesthetics was measured by three items associated with the building’s appearance (BA1), colors (BA2), and shape (BA3). External Orientation was measured by four items associated with the existence of signposts (OE1), the ease of finding it (OE2), finding its entrance (OE3), and difficulty of finding it (OE4).

Finally, five constructs—Lighting, Spatial Physical Comfort, Air Quality, Internal Orientation, and Quietness—formed Internal Spaces. Lighting was measured by three items associated with the sunlight (L1), the size (L2), and the number of windows (L3). Spatial Physical Comfort was measured by eleven items associated with furnishing conditions (SPC1), appearance (SPC2), and quality (SPC3), the maintenance of walls, floors, and ceilings (SPC4), their colors (SPC5), condition (SPC6), and appearance (SPC7), window cleanliness (SPC8), seats’ comfort (SPC9), their number (SPC10), and the equipment of waiting area (SPC11)). Air Quality was measured by four items associated with room temperature (AQ1), air exchange (AQ2), humidity (AQ3), and air freshness (AQ4). Internal Orientation was measured by four items associated with the signposts in the institution (OI1), and their number (OI2), location(s) of information point(s) (OI3), and the ease of finding it (them) (OI4). Quietness was measured by four items associated with the level of quietness (Q1), dins and screams (Q2), the level of noise (Q3), and its frequency (Q4).

For measuring green healthcare practices, we used the scale presented in the research of González-Viralta et al. [38], which was adapted to the context of healthcare institutions. The scale consisted of four items related to the patients’ perceptions of the Institute for Student Health Protection in Novi Sad regarding its compliance with environmental standards defined by law (GP1), effort to improve green practices (GP2), concerns for respecting and protecting the environment (GP3), and the improvement of the general well-being of society (GP4).

The construct of environmental knowledge was evaluated in line with the scale used by Myung [53]. It included five items associated with patients’ knowledge related to the preservation of the environment (EK1), global warming (EK2), acid rain (EK3), the problem of ozone depletion (EK4), and decomposition of plastic bags (EK5).

When it comes to patient satisfaction, following Senić and Marinković [23], we applied a three-item scale with a focus on overall satisfaction (S1), patients’ expectations (S2), and the proximity to ideal service (S3).

### 3.3. Statistical Analysis

**Figure 2:**
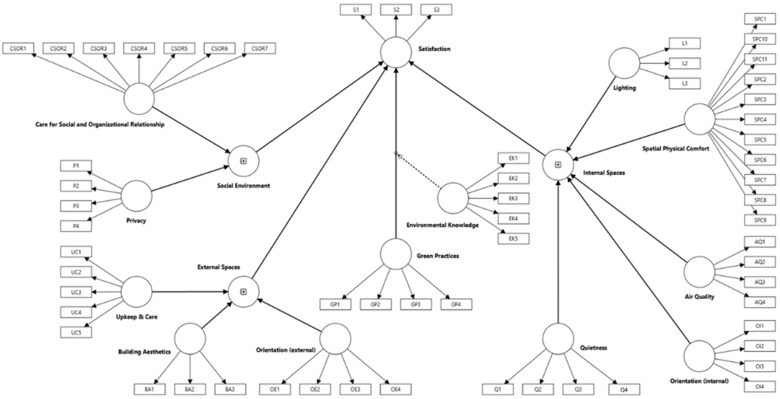
Research model.

The research model is presented in Figure 2. It consists of three hierarchical reflective–formative constructs (Social Environment, External Spaces, and Internal Spaces) and three reflective constructs (green practices, environmental knowledge, and patient satisfaction).

Each hierarchical construct was formed by a set of reflective lower-order constructs: Social Environment was formed by Care for Social and Organizational Relationship, and Privacy; External Spaces by Upkeep and Care, Building Aesthetics, and Orientation (external); and Internal Spaces by Lighting, Spatial Physical Comfort, Air Quality, Orientation (internal), and Quietness. The application of hierarchical reflective–formative modeling was based on the use of a repeated indicator approach [54], meaning that higher-order constructs were specified with items from their corresponding lower-order constructs.

As all constructs are latent (unobservable), the PLS-SEM, i.e., the partial least squares structural equation modeling, was used for testing the model and analyzing relations between them. This approach represents the advanced statistical technique of second-generation that is well suited for complex models with many indicators [52], and is particularly appropriate for examining hierarchical (higher-order) constructs [54].

Following Hair et al. [52], when testing reflective constructs (including the lower-order ones), the emphasis was on the analysis of the following:

- Indicator reliability (outer loadings);
- Internal consistency reliability (Composite Reliability—CR, and Cronbach’s α);
- Convergent validity (Average Variance Extracted—AVE); and
- Discriminant validity (the application of the Fornell–Larcker and the HTMT criterion).

According to the same authors [52], the evaluation of higher-order (formative) constructs involved the examination of convergent validity, multicollinearity statistics, and path coefficients. Hereby, when it comes to the convergent validity, we applied the redundancy analysis and introduced three global items—one for each formative higher-order construct.

To test the hypotheses, path coefficients and their significance levels were analyzed, followed by an examination of their ƒ^2^ (effect sizes). Effect size values of 0.02, 0.15, and 0.35 indicate small, medium, and large effects, respectively [52].

In addition, considering that all data were obtained from the same source, i.e., the same students evaluated all statements, there was a potential risk of common method bias. Therefore, in addition to ensuring the anonymity of the survey, Harman’s one-factor test (in MS Excel) was applied to assess the presence of such bias. If the first factor explains less than 50% of the total variance, common method bias does not appear to be an important issue [55].

## 4. Results

Negatively formulated items were reverse-coded in the Excel file during data preparation. Thereafter, the model was tested, beginning with reflective constructs and then proceeding to formative constructs. Indicators CSOR7, P2, P3, UC1, UC2, OE1, SPC8, SPC10, SPC11, AQ1, OI1, OI2, EK1, EK2, and EK5 had outer loadings lower than 0.70, because of which they were eliminated. The analysis was repeated, after which we obtained the results presented in Table 1.

Outer loadings higher than 0.70, and values of CR, AVE, and Cronbach’s α above 0.70, 0.50, and 0.70 respectively, represent satisfactory reliability and convergent validity measures. It should be noted that, after the elimination process, only two indicators remained for each of the three constructs (Privacy, Orientation (internal), and environmental knowledge). However, given the acceptable levels of reliability and validity, and considering their reflective nature, the remaining indicators can be considered adequate measures of the respective constructs [56].

Based on the results of Harman’s one-factor test, in which the first factor accounted for approximately 32% of the total variance (well below the 50% threshold), common method bias was not considered a serious concern.

Regarding discriminant validity, the values related to the Fornell–Larcker criterion were satisfactory (Table 2), as the square root of the AVE for each construct (on the diagonal) was greater than the corresponding inter-construct correlations (off-diagonal values).

The same applies to the HTMT, given that all values were below 0.85 for each construct pair (Table 3).

Concerning formative constructs (Table 4), the analysis of path coefficients has shown that lower-order constructs significantly (at *p* < 0.001) formed the corresponding higher-order ones. In addition, VIF (variance inflation factor) values lower than 3 indicated no multicollinearity issues.

Three new models were created for the redundancy analysis, each consisting of one formative (higher-order) construct connected with corresponding lower-order constructs and a new global single-item construct. When it comes to Social Environment and Internal Spaces, the path coefficients from higher-order constructs to single-item ones were above 0.70 (0.721 and 0.709, respectively), while their R^2^ values were higher than 0.50 (0.519 and 0.503, respectively), confirming their convergent validity. On the other hand, in the case of External Spaces, the previously mentioned indicators were just below the threshold values of 0.70 for path coefficient (0.685) and 0.50 for R^2^ (0.470). As these gaps are minor, we accepted the obtained results and continued the analysis.

On the inner model level, the R^2^ for patient satisfaction equaled 0.615, and the Q^2^ predicted value was above 0. All path coefficients were positive regarding the effects on patient satisfaction, with VIF values lower than 3 (Table 5). However, significant ones (with *p*-values lower than 0.001) were recorded for Social Environment and green practices, supporting hypotheses H3 and H4. In both these cases, ƒ^2^ values were above the small effect threshold, with Social Environment showing medium effect, and green practices a small effect. The first two hypotheses (H1 and H2), as well as the one associated with the moderating effect (H5), stayed unconfirmed, bearing in mind that *p*-values related to the effects of External Spaces and Internal Spaces, as well as to the effect of the interaction term (environmental knowledge × green practices) on patient satisfaction were higher than 0.05. For these relations, ƒ^2^ values were below 0.02, indicating no effect.

## 5. Discussion

Among the four determinants of patient satisfaction, the largest effect was recorded for the Social Environment. In addition, it was the only aspect of the environment that significantly affected patient satisfaction. Its positive influence implies that the increase in the quality of the social relationship with the patient would lead to an increase in the level of his (her) satisfaction. This is in line with the research of Senić and Marinković [23], who found the largest positive impact of personal relationships on patient satisfaction. Moreover, Andaleeb [22], among others, found that patient satisfaction was positively and significantly affected by the communication and responsiveness of the hospital staff. Bearing in mind that healthcare services are strongly based on human factors and direct interaction with patients, the social aspect of the environment should be continuously monitored and nurtured in order to satisfy patients’ expectations. Hence, the courtesy of the healthcare workers (as also proven in some previous research [57,58]), their understanding of patients, and the organizational atmosphere represent important elements in improving patient satisfaction.

As previously mentioned, the other two aspects of the environment—Internal Spaces and External Spaces—did not significantly influence patient satisfaction. There can be several reasons for this. The first one relates to the spatial limitations of the Institute for Student Health Protection in Novi Sad, which is located within the University Campus. The second one refers to the type of healthcare services provided in this institution, which, despite being diverse, usually do not require a long stay for patients. Due to these relatively short stays, students may not pay much attention to the spatial elements. In addition, because of its position within the Campus, there is a possibility that they have become familiar with the environment.

Besides environmental factors, the subject of the analysis was the impact of green hospital practices on patient satisfaction. Its positive and significant effect (direct or indirect) was also found in similar studies [25,36]. Hence, the implementation of green practices, their improvement, and compliance with environmental standards may increase the satisfaction of service users in the healthcare sector, which can be of special importance, bearing in mind that those services significantly rely on different natural resources [25]. As the research results have shown, there was no significant moderating effect of environmental knowledge. This can be attributed to the sampled population—students—who, as young and educated individuals, are generally familiar with environmental issues [59,60]. The reason may also be in the measurement process, bearing in mind the simplicity of the items used for this purpose, as well as in potential issues related to a homogeneous sample.

## 6. Conclusions

### 6.1. Managerial Implications

The research finding related to the significant effect of the Social Environment is particularly relevant from a managerial perspective, and thus, several managerial implications can be derived. It is obvious that the Social Environment, i.e., social relations with patients, is a source of greater patient satisfaction. Satisfied employees will foster customers’ satisfaction. This kind of environment is built through a serious human resource management (HRM) approach which includes adequate staffing, organization, training, team building, compensation, and motivation practices. When it comes to staffing, it is important to attract and select the type of person who is dedicated to serving people. Those are employees with a strong career anchor in security, stability, and service, like nurses for example [61]. It is necessary to investigate their personality, their dedication, to select those who are truly committed to patients care. Of course, the starting point is to have employees with adequate competences in providing medical care. Organization of personnel is another aspect that leads to greater patients’ satisfaction and success of one healthcare organization. The organization of personnel in a healthcare setting plays a critical role in patient satisfaction and the overall success of the organization. Effective personnel management ensures that healthcare providers work efficiently, deliver high-quality care, and create a positive patient experience. Proper staff allocation ensures that patients receive timely attention and treatment. Interdisciplinary collaboration between doctors, nurses, and support staff leads to better decision-making and treatment outcomes. Reducing wait times and streamlining workflows enhance patient trust and satisfaction. After staffing and organization, proper training not only for providing care, but for emotional intelligence, is mandatory too. Patients’ satisfaction is not only affected by the medical treatment they receive, but by the relationship they feel during the whole process. That is why employees need to be equipped with knowledge and skills for emotional support, understanding, care, etc. Also, their internal relationships and work atmosphere is crucial for delivering good service. If employees are working in a healthy work environment and have good relations between them, they will be more satisfied at work and dedicate their effort towards their service users. In the end, motivational approach and compensation management are crucial for retaining the best employees and for fostering a good organizational climate and culture. Compensation as a reward for employees’ work includes monetary and nonmonetary rewards for work and behavior of employees. Therefore, compensation mechanisms need to reward performances, but also the kind of behavior that leads to greater satisfaction of patients. From the aspect of HRM, only concerning all those elements, healthcare organization can retain and increase good services and patients’ satisfaction.

### 6.2. Financial Implications

The implementation of green practices in healthcare can lead to significant financial challenges, but can also provide long-term economic benefits. Initial investments in energy-efficient infrastructure, waste reduction programs, and sustainable procurement require substantial funding, which can be a barrier for many institutions. However, these costs can be mitigated through governmental incentives, green financing programs, and long-term cost efficiencies. Policy frameworks, including tax benefits, grants, and low-interest loans, support organizations in adopting sustainable practices. Hospitals, as significant energy consumers, should actively promote policies that enhance energy efficiency and support climate initiatives. By proactively adopting sustainable measures, healthcare institutions can optimize energy use, reduce operational costs, and avoid potential regulatory fines while strengthening their public image [62]. Strategic investments in green infrastructure, such as energy-efficient buildings and renewable energy systems, can contribute to increased property values and facilitate access to funding opportunities aligned with Environmental, Social, and Governance (ESG) principles. Institutions implementing eco-friendly measures typically achieve significant reductions in energy and resource consumption. Government incentives play a crucial role in this transition, particularly tax breaks for energy-efficient structures that help offset initial investment barriers [63]. Green procurement strategies, such as using biodegradable materials and waste minimization programs, further enhance cost savings while demonstrating environmental responsibility. The combination of operational efficiencies, reputational benefits, and regulatory compliance makes sustainable healthcare practices a strategically sound investment. Through public–private collaborations and innovative financing models, healthcare organizations can implement environmentally responsible solutions that deliver both financial and ecological returns, ultimately contributing to a more sustainable healthcare system.

### 6.3. Limitations and Future Research

This study has several limitations that should be acknowledged. The research was based on patients’ perceptions, which are subjective in nature. While such an approach aligns with the principles of service marketing, it would be beneficial to supplement these findings with objective indicators, especially for constructs where such measures are applicable. Furthermore, this study focused solely on a student population, which limits the generalizability of the results. Therefore, future research could include participants (patients) from other healthcare institutions, focusing on different population groups. In future studies, the proposed model could be extended by incorporating additional moderating effects. Among them, some demographic characteristics (such as gender) could be considered. Finally, it would also be interesting to explore patients’ behavioral intentions.

## Figures and Tables

**Table 1:** Reflective constructs: outer loadings, CR, and AVE.

Indicators	Outer Loadings	CR	AVE	Cronbach’s α
Care for Social and Organizational Relationship (CSOR)		0.903	0.609	0.871
(CSOR1) In the Institute for Student Health Protection, people receive a nice welcome from staff.	0.815			
(CSOR2) In the Institute for Student Health Protection, doctors are generally not very understanding toward patients.	0.823			
(CSOR3) In the Institute for Student Health Protection, nurses are generally not very understanding toward patients.	0.793			
(CSOR4) In the Institute for Student Health Protection, doctors generally provide poor information on medical examinations, therapies, and interventions.	0.746			
(CSOR5) In the Institute for Student Health Protection, there is a good cooperative atmosphere among staff members.	0.709			
(CSOR6) The Institute for Student Health Protection is poorly organized.	0.791			
Privacy (P)		0.929	0.867	0.847
(P1) In the Institute for Student Health Protection, you can talk to staff about delicate issues without being overheard by others.	0.929			
(P4) In the Institute for Student Health Protection, people can have their own privacy.	0.933			
Upkeep and Care (UC)		0.888	0.725	0.809
(UC3) In the external area of the Institute for Student Health Protection, paths and sidewalks are in good condition.	0.845			
(UC4) The external area of the Institute for Student Health Protection is well-kept.	0.912			
(UC5) The external area of the Institute for Student Health Protection is not very clean.	0.793			
Building Aesthetics (BA)		0.898	0.747	0.830
(BA1) From the outside, the building of the Institute for Student Health Protection is nice.	0.824			
(BA2) From the outside, the colors of the building of the Institute for Student Health Protection are unpleasant.	0.885			
(BA3) From the outside, the shape of the building of the Institute for Student Health Protection is unpleasant.	0.882			
Orientation (external) (OE)		0.913	0.779	0.858
(OE2) It is easy to find the Institute for Student Health Protection.	0.903			
(OE3) It is easy to find the entrance of the Institute for Student Health Protection.	0.875			
(OE4) It is difficult to get oriented and find the Institute for Student Health Protection.	0.869			
Lighting (L)		0.829	0.619	0.704
(L1) There is not enough sunlight in the Institute for Student Health Protection.	0.840			
(L2) The Institute for Student Health Protection has large windows.	0.726			
(L3) The Institute for Student Health Protection needs more windows.	0.791			
Spatial Physical Comfort (SPC)		0.928	0.618	0.912
(SPC1) In the Institute for Student Health Protection, furnishings are in good condition.	0.805			
(SPC2) In the Institute for Student Health Protection, furnishings are unpleasant.	0.796			
(SPC3) In the Institute for Student Health Protection, the quality of furnishings is good.	0.826			
(SPC4) In the Institute for Student Health Protection, walls, floors, and ceilings are well kept.	0.814			
(SPC5) In the Institute for Student Health Protection, walls, floors, and ceilings have nice colors.	0.777			
(SPC6) In the Institute for Student Health Protection, walls, floors, and ceilings are in poor condition.	0.776			
(SPC7) In the Institute for Student Health Protection, walls, floors, and ceilings are unpleasant.	0.771			
(SPC9) In the Institute for Student Health Protection, seats are uncomfortable.	0.722			
Air Quality (AQ)		0.897	0.743	0.827
(AQ2) In the Institute for Student Health Protection, the air exchange from outside is adequate.	0.881			
(AQ3) In the Institute for Student Health Protection, air humidity is adequate (neither too wet nor too dry).	0.836			
(AQ4) In the Institute for Student Health Protection, the air is not fresh.	0.869			
Orientation (internal) (OI)		0.918	0.849	0.822
(OI3) In the Institute for Student Health Protection, information point(s) is (are) badly located.	0.925			
(OI4) In the Institute for Student Health Protection, you can easily find information point(s).	0.918			
Quietness (Q)		0.914	0.728	0.874
(Q1) In the Institute for Student Health Protection, there is enough quietness.	0.870			
(Q2) In the Institute for Student Health Protection, you can hear dins and screams.	0.898			
(Q3) In the Institute for Student Health Protection, you can hear little noise from outside.	0.872			
(Q4) In the Institute for Student Health Protection, you can often hear noise from the outside.	0.768			
Green Practices (GP)		0.909	0.715	0.868
(GP1) The healthcare institute respects the environmental norms defined in the law when carrying out its activities.	0.836			
(GP2) The healthcare institute tries to improve its green practices.	0.837			
(GP3) The healthcare institute is concerned with respecting and protecting the natural environment.	0.893			
(GP4) The healthcare institute is concerned with improving the general well-being of society.	0.815			
Environmental Knowledge (EK)		0.880	0.787	0.762
(EK3) I know the meaning of “acid rain”.	0.964			
(EK4) I know what the problem of ozone depletion is.	0.802			
Satisfaction (S)		0.928	0.811	0.884
(S1) Overall, I am satisfied with the services of the Institute for Student Health Protection.	0.891			
(S2) The overall organization of the Institute for Student Health Protection is above my expectations.	0.900			
(S3) The Institute for Student Health Protection is close to delivering ideal service.	0.911			

**Table 2:** Reflective constructs: Fornell–Larcker criterion.

	AQ	BA	CSOR	EK	GP	L	OE	OI	P	Q	S	SPC	UC
**AQ**	**0.862**												
**BA**	0.307	**0.864**											
**CSOR**	0.472	0.381	**0.780**										
**EK**	0.036	−0.048	−0.029	**0.887**									
**GP**	0.512	0.350	0.623	−0.072	**0.846**								
**L**	0.409	0.307	0.320	−0.038	0.280	**0.787**							
**OE**	0.329	0.363	0.305	0.031	0.210	0.210	**0.882**						
**OI**	0.448	0.277	0.427	0.015	0.410	0.231	0.318	**0.921**					
**P**	0.376	0.335	0.582	−0.008	0.531	0.226	0.286	0.333	**0.931**				
**Q**	0.481	0.273	0.445	0.074	0.440	0.204	0.194	0.453	0.504	**0.853**			
**S**	0.460	0.335	0.722	−0.100	0.666	0.301	0.323	0.391	0.557	0.358	**0.901**		
**SPC**	0.524	0.588	0.534	−0.060	0.561	0.400	0.303	0.356	0.427	0.350	0.532	**0.786**	
**UC**	0.413	0.395	0.444	−0.004	0.348	0.230	0.252	0.366	0.344	0.410	0.315	0.386	**0.852**

Air Quality (AQ), Building Aesthetics (BA), Care for Social and Organizational Relationship (CSOR), Environmental Knowledge (EK), Green Practices (GP), Lighting (L), Orientation (external) (OE), Orientation (internal) (OI), Privacy (P), Quietness (Q), Satisfaction (S), Spatial Physical Comfort (SPC), Upkeep and Care (UC).

**Table 3:** Reflective constructs: HTMT criterion.

	AQ	BA	CSOR	EK	GP	L	OE	OI	S	P	Q	SPC	UC
**AQ**													
**BA**	0.369												
**CSOR**	0.556	0.447											
**EK**	0.068	0.082	0.062										
**GP**	0.603	0.410	0.704	0.104									
**L**	0.517	0.387	0.372	0.065	0.323								
**OE**	0.389	0.426	0.352	0.072	0.238	0.267							
**OI**	0.542	0.333	0.503	0.083	0.482	0.283	0.377						
**S**	0.537	0.390	0.822	0.135	0.747	0.356	0.369	0.459					
**P**	0.448	0.399	0.678	0.036	0.608	0.268	0.333	0.398	0.643				
**Q**	0.564	0.316	0.509	0.110	0.497	0.233	0.222	0.533	0.407	0.584			
**SPC**	0.602	0.678	0.598	0.088	0.629	0.473	0.339	0.409	0.593	0.485	0.390		
**UC**	0.505	0.482	0.530	0.081	0.410	0.282	0.301	0.447	0.372	0.415	0.486	0.448	

Air Quality (AQ), Building Aesthetics (BA), Care for Social and Organizational Relationship (CSOR), Environmental Knowledge (EK), Green Practices (GP), Lighting (L), Orientation (external) (OE), Orientation (internal) (OI), Satisfaction (S), Privacy (P), Quietness (Q), Spatial Physical Comfort (SPC), Upkeep and Care (UC).

**Table 4:** Formative constructs: path coefficients and VIF.

Lower-Order Constructs	Higher-Order Constructs	Path Coefficients	*p* Values	VIF
Air Quality	Internal Spaces	0.227	*p* < 0.001	1.781
Lighting		0.124	*p* < 0.001	1.275
Orientation (internal)		0.137	*p* < 0.001	1.401
Quietness		0.247	*p* < 0.001	1.445
Spatial Physical Comfort		0.585	*p* < 0.001	1.509
Privacy	Social Environment	0.298	*p* < 0.001	1.513
Care for Social and Organizational Relationships		0.797	*p* < 0.001	1.513
Building Aesthetics	External Spaces	0.472	*p* < 0.001	1.299
Orientation (external)		0.452	*p* < 0.001	1.171
Upkeep and Care		0.413	*p* < 0.001	1.205

**Table 5:** Inner mode: path coefficients and VIF.

Relations	Path Coeff.	*p* Values	ƒ^2^	VIF	Hypotheses
External Spaces → Satisfaction	0.003	0.949	0.000	1.755	H_1_ Rejected
Internal Spaces → Satisfaction	0.084	0.187	0.007	2.600	H_2_ Rejected
Social Environment → Satisfaction	0.502	*p* < 0.001	0.298	2.203	H_3_ Supported
Green Practices → Satisfaction	0.273	*p* < 0.001	0.089	2.169	H_4_ Supported
Env. Knowledge x Green Practices → Satisfaction	0.011	0.766	0.000	1.147	H_5_ Rejected

## Data Availability

Not all data are contained in the article. Data sharing is not applicable to this article.

## References

[1] Gonzalez M.E. (2019). Improving customer satisfaction of a healthcare facility: Reading the customers’ needs. Benchmarking Int. J..

[2] Baashar Y., Alhussian H., Patel A., Alkawsi G., Alzahrani A.I., Alfarraj O., Hayder G. (2020). Customer relationship management systems (CRMS) in the healthcare environment: A systematic literature review. Comput. Stand. Interfaces.

[3] Kurtuluş S.A., Cengiz E. (2022). Customer experience in healthcare: Literature review. Istanb. Bus. Res..

[4] Park S.Y., Yun G.W., Friedman S., Hill K., Coppes M.J. (2022). Patient-centered care and healthcare consumerism in online healthcare service advertisements: A positioning analysis. J. Patient Exp..

[5] Delaney L.J. (2018). Patient-centred care as an approach to improving health care in Australia. Collegian.

[6] Ross C.K., Frommelt G., Hazelwood L., Chang R.W. (1987). The role of expectations in patient satisfaction with medical care. Mark. Health Serv..

[7] Mutingi M., Gwangwava N., Mutingi M. (2018). Towards a customer-centric framework for evaluation of e-health service quality. E-Manufacturing and E-Service Strategies in Contemporary Organizations.

[8] Calculli C., D’Uggento A.M., Labarile A., Ribecco N. (2021). Evaluating people’s awareness about climate changes and environmental issues: A case study. J. Clean. Prod..

[9] Marimuthu M., Paulose H. (2016). Emergence of sustainability based approaches in healthcare: Expanding research and practice. Procedia-Soc. Behav. Sci..

[10] Bosco F., Di Gerio C., Fiorani G., Stola G. (2024). How to manage sustainability in healthcare organizations? A processing map to include the ESG strategy. J. Public Budg. Account. Financ. Manag..

[11] Thomas A., Ma S., Ur Rehman A., Usmani Y.S. (2023). Green operation strategies in healthcare for enhanced quality of life. Healthcare.

[12] Berniak-Woźny J., Rataj M. (2023). Towards green and sustainable healthcare: A literature review and research agenda for green leadership in the healthcare sector. Int. J. Environ. Res. Public Health.

[13] Soares A.L., Buttigieg S.C., Bak B., McFadden S., Hughes C., McClure P., Couto J.G., Bravo I. (2023). A review of the applicability of current green practices in healthcare facilities. Int. J. Health Policy Manag..

[14] World Health Organization (2024). Health-Care Waste.

[15] Orsini L.P., Landi S., Leardini C., Veronesi G. (2024). Towards greener hospitals: The effect of green organisational practices on climate change mitigation performance. J. Clean. Prod..

[16] Dhillon V.S., Kaur D. (2015). Green hospital and climate change: Their interrelationship and the way forward. J. Clin. Diagn. Res..

[17] Al-Shehri S.N. (2002). Healthy students–healthy nation. J. Fam. Community Med..

[18] Stewart-Brown S., Evans J., Patterson J., Petersen S., Doll H., Balding J., Regis D. (2000). The health of students in institutes of higher education: An important and neglected public health problem?. J. Public Health.

[19] Linder-Pelz S. (1982). Toward a theory of patient satisfaction. Soc. Sci. Med..

[20] Williams B. (1994). Patient satisfaction: A valid concept?. Soc. Sci. Med..

[21] Sitzia J., Wood N. (1997). Patient satisfaction: A review of issues and concepts. Soc. Sci. Med..

[22] Andaleeb S.S. (2001). Service quality perceptions and patient satisfaction: A study of hospitals in a developing country. Soc. Sci. Med..

[23] Senić V., Marinković V. (2013). Patient care, satisfaction and service quality in health care. Int. J. Consum. Stud..

[24] Rauf A., Muhammad N., Mahmood H., Yen Y.Y. (2024). The influence of healthcare service quality on patients’ satisfaction in urban areas: The case of Pakistan. Heliyon.

[25] Chakraborty S., Sashikala P., Roy S. (2022). Green–agile practices as drivers for patient satisfaction—An empirical study. Int. J. Healthc. Manag..

[26] Manzoor F., Wei L., Hussain A., Asif M., Shah S.I.A. (2019). Patient satisfaction with health care services; an application of physician’s behavior as a moderator. Int. J. Environ. Res. Public Health.

[27] Andrade C., Lima M.L., Fornara F., Bonaiuto M. (2012). Users’ views of hospital environmental quality: Validation of the perceived hospital environment quality indicators (PHEQIs). J. Environ. Psychol..

[28] Fornara F., Bonaiuto M., Bonnes M. (2006). Perceived hospital environment quality indicators: A study of orthopaedic units. J. Environ. Psychol..

[29] Shadmi E. (2013). Quality of hospital to community care transitions: The experience of minority patients. Int. J. Qual. Health Care.

[30] Andrade C.C., Lima M.L., Pereira C.R., Fornara F., Bonaiuto M. (2013). Inpatients’ and outpatients’ satisfaction: The mediating role of perceived quality of physical and social environment. Health Place.

[31] LaVela S.L., Etingen B., Hill J.N., Miskevics S. (2016). Patient perceptions of the environment of care in which their healthcare is delivered. HERD Health Environ. Res. Des. J..

[32] Zisberg A., Syn-Hershko A. (2016). Factors related to the mobility of hospitalized older adults: A prospective cohort study. Geriatr. Nurs..

[33] Mohammad Moradi A., Hosseini S.B., Shamloo G. (2018). Evaluating the impact of environmental quality indicators on the degree of humanization in healing environments. Space Ontol. Int. J..

[34] Manca S., Bonaiuto M., Fornara F. (2023). Perceived hospital environment quality indicators: The case of healthcare places for terminal patients. Buildings.

[35] Edris N., Bashir F., Zeleke B. (2024). Impacts of hospitals users’ characteristics on perceptions of the physical environment. Heliyon.

[36] Mahrinasari M.S., Haseeb M., Bangsawan S., Sabri M.F., Daud N.M. (2023). Effects of Green Operational Practices and E-CRM on Patient Satisfaction among Indonesian Hospitals: Exploring the Moderating Role of Green Social Influence. Oper. Res. Eng. Sci. Theory Appl..

[37] Moise M.S., Gil-Saura I., Ruiz-Molina M.E. (2021). “Green” practices as antecedents of functional value, guest satisfaction and loyalty. J. Hosp. Tour. Insights.

[38] González-Viralta D., Veas-González I., Egaña-Bruna F., Vidal-Silva C., Delgado-Bello C., Pezoa-Fuentes C. (2023). Positive effects of green practices on the consumers’ satisfaction, loyalty, word-of-mouth, and willingness to pay. Heliyon.

[39] Mai K.N., Nhan D.H., Nguyen P.T.M. (2023). Empirical study of green practices fostering customers’ willingness to consume via customer behaviors: The case of green restaurants in Ho Chi Minh City of Vietnam. Sustainability.

[40] Chaturvedi P., Kulshreshtha K., Tripathi V., Agnihotri D. (2024). Investigating the impact of restaurants’ sustainable practices on consumers’ satisfaction and revisit intentions: A study on leading green restaurants. Asia-Pac. J. Bus. Adm..

[41] Fryxell G.E., Lo C.W. (2003). The influence of environmental knowledge and values on managerial behaviours on behalf of the environment: An empirical examination of managers in China. J. Bus. Ethics.

[42] Vicente-Molina M.A., Fernández-Sáinz A., Izagirre-Olaizola J. (2013). Environmental knowledge and other variables affecting pro-environmental behaviour: Comparison of university students from emerging and advanced countries. J. Clean. Prod..

[43] Fu L., Zhang Y., Bai Y. (2018). Pro-environmental awareness and behaviors on campus: Evidence from Tianjin, China. Eurasia J. Math. Sci. Technol. Educ..

[44] Liu P., Teng M., Han C. (2020). How does environmental knowledge translate into pro-environmental behaviors?: The mediating role of environmental attitudes and behavioral intentions. Sci. Total Environ..

[45] Fraj-Andrés E., Martínez-Salinas E. (2007). Impact of environmental knowledge on ecological consumer behaviour: An empirical analysis. J. Int. Consum. Mark..

[46] Hamzah M.I., Tanwir N.S. (2021). Do pro-environmental factors lead to purchase intention of hybrid vehicles? The moderating effects of environmental knowledge. J. Clean. Prod..

[47] Park J., Ryu Y., Kim Y. (2024). Factors influencing air passengers’ intention to purchase voluntary carbon offsetting programs: The moderating role of environmental knowledge. J. Air Transp. Manag..

[48] Issock Issock P.B., Mpinganjira M., Roberts-Lombard M. (2020). Modelling green customer loyalty and positive word of mouth: Can environmental knowledge make the difference in an emerging market?. Int. J. Emerg. Mark..

[49] Statistical Office of the Republic of Serbia (2021). Statistical Yearbook of the Republic of Serbia. Belgrade. https://publikacije.stat.gov.rs/G2021/PdfE/G20212054.pdf.

[50] Statistical Office of the Republic of Serbia (2022). Statistical Yearbook of the Republic of Serbia. Belgrade. https://publikacije.stat.gov.rs/G2022/pdf/G20222055.pdf.

[51] Statistical Office of the Republic of Serbia (2023). Statistical Yearbook of the Republic of Serbia. Belgrade. https://publikacije.stat.gov.rs/G2023/Pdf/G20232056.pdf.

[52] Hair J.F., Hult G.T.M., Ringle C.M., Sarstedt M. (2017). A Primer on Partial Least Squares Structural Equation Modeling.

[53] Myung E. (2018). Environmental knowledge, attitudes, and willingness to pay for environmentally friendly meetings–An exploratory study. J. Hosp. Tour. Manag..

[54] Becker J.M., Klein K., Wetzels M. (2012). Hierarchical Latent Variable Models in PLS-SEM: Guidelines for Using Reflective-Formative Type Models. Long. Range Plan..

[55] Liang T.P., Lin Y.L., Shiau W.L., Chen S.F. (2021). Investigating common method bias via an EEG study of the flow experience in website design. J. Electron. Commer. Res..

[56] Fleuren B., van Amelsvoort L., Zijlstra F.R.H., de Grip A., Kant I. (2018). Handling the reflectiveformative measurement conundrum: A practical illustration based on sustainable employability. J. Clin. Epidemiol..

[57] Van de Ven A.H. (2014). What matters most to patients? Participative provider care and staff courtesy. Patient Exp. J..

[58] Asres A.W., Hunegnaw W.A., Ferede A.G., Denekew H.T. (2020). Assessment of patient satisfaction and associated factors in an outpatient department at Dangila primary hospital, Awi zone, Northwest Ethiopia, 2018. Glob. Secur. Health Sci. Policy.

[59] Lai C.K.M., Cheng E.W.L. (2016). Green purchase behavior of undergraduate students in Hong Kong. Soc. Sci. J..

[60] Kumar B., Manrai A.K., Manrai L.A. (2017). Purchasing behaviour for environmentally sustainable products: A conceptual framework and empirical study. J. Retail. Consum. Serv..

[61] Nascimento F.P.B., Sousa K.H.J.F., Tomaz A.P.K.D.A., Tracera G.M.P., Santos K.M.D., Oliveira E.B.D., Zeitoune R.C.G. (2021). Nursing career anchors and professional exercise: Is there alignment?. Rev. Bras. Enferm..

[62] Dion H., Evans M., Farrell P. (2023). Hospitals management transformative initiatives; towards energy efficiency and environmental sustainability in healthcare facilities. J. Eng. Des. Technol..

[63] Khahro S.H., Kumar D., Siddiqui F.H., Ali T.H., Raza M.S., Khoso A.R. (2021). Optimizing Energy Use, Cost and Carbon Emission through Building Information Modelling and a Sustainability Approach: A Case-Study of a Hospital Building. Sustainability.

